# On the Reaction Pathways and Growth Mechanisms of LiNbO_3_ Nanocrystals from the Non-Aqueous Solvothermal Alkoxide Route

**DOI:** 10.3390/nano11010154

**Published:** 2021-01-09

**Authors:** Mathias Urbain, Florian Riporto, Sandrine Beauquis, Virginie Monnier, Jean-Christophe Marty, Christine Galez, Christiane Durand, Yann Chevolot, Ronan Le Dantec, Yannick Mugnier

**Affiliations:** 1 SYMME, University of Savoie Mont Blanc, F-74000 Annecy, France; mathias.urbain45@gmail.com (M.U.); florian.riporto@gmail.com (F.R.); Sandrine.Beauquis@univ-smb.fr (S.B.); Jean-Christophe.Marty@univ-smb.fr (J.-C.M.); Christine.Galez@univ-smb.fr (C.G.); Christiane.Durand@univ-smb.fr (C.D.); Ronan.le-Dantec@univ-smb.fr (R.L.D.); 2 Institut des Nanotechnologies de Lyon (INL), UMR CNRS 5270, Ecole Centrale de Lyon, Université de Lyon, F-69134 Ecully CEDEX, France; Virginie.Monnier@ec-lyon.fr (V.M.); Yann.Chevolot@ec-lyon.fr (Y.C.)

**Keywords:** lithium niobate nanocrystals, alkoxide precursors, reaction pathways and growth mechanisms, non-aqueous solvothermal conditions, size and shape control

## Abstract

Phase-pure, highly crystalline sub-50 nm LiNbO_3_ nanocrystals were prepared from a non-aqueous solvothermal process for 72 h at 230 °C and a commercial precursor solution of mixed lithium niobium ethoxide in its parent alcohol. A systematic variation of the reaction medium composition with the addition of different amounts of co-solvent including butanol, 1,3-propanediol, 1,4-butanediol, and 1,5-pentanediol resulted in the formation of nanocrystals of adjustable mean size and shape anisotropy, as demonstrated from XRD measurements and TEM imaging. Colloidal stability of ethanol- and water-based suspensions was evaluated from dynamic light scattering (DLS)/zeta potential studies and correlated with FTIR data. Thanks to the evolution in the nanocrystal size and shape distribution we observed, as well as to the available literature on the alkoxide chemistry, the reaction pathways and growth mechanisms were finally discussed with a special attention on the monomer formation rate, leading to the nucleation step. The polar, non-perovskite crystalline structure of LiNbO_3_ was also evidenced to play a major role in the nanocrystal shape anisotropy.

## 1. Introduction

Among the optically active multifunctional materials, the non-perovskite crystalline structure of lithium niobate (LiNbO_3_, LN) has attracted a considerable research interest due to its large transparency range [1], stoichiometry-dependent electro-optic response [2], decent non-linear optical (NLO) properties [3], and its tunable photorefractivity and luminescence, since the structure has proven to be a good host for several metal and rare-earth fluorescent ions [4]. LN also possesses unique acoustic and piezoelectric responses, making this material suitable for a wide range of applications including optical modulators, frequency converters, acoustic transducers, and surface acoustic wave devices [5,6]. Wafers of LN are today available worldwide and are most often employed as band-pass filters in mobile phones and in other consumer electronic applications. In the integrated optics field, a series of new promising photonic devices have also recently emerged, making use of its electro-optic and second harmonic generation (SHG) properties [7,8,9]. On the other hand, with the current trend in the miniaturization of smart devices, synthesis and characterizations of noncentrosymmetric oxides (NCO) at the nanoscale have both been driven in the last 20 years by fundamental and technological standpoints, as already evidenced in LN and in several perovskite structures such as BaTiO_3_ and its derivatives [10,11,12]. In terms of SHG properties, deviations from the bulk response have, for instance, only been noticed in BaTiO_3_ [13] and LN [14], but below a typical nanocrystal size of ≈20 nm. In the 20–150 nm size regime [15], one of the key benefits resulting from the reduced dimensions is very likely the absence of phase-matching conditions allowing the simultaneous emission from NCO nanocrystals of multiple NLO signals [16,17]. Applications of these nonlinear point-like dipoles as exogenous markers in the biomedical imaging field have thus allowed circumventing some of the limits of the classical fluorescence-based microscopy. The imaging depth can be increased [18]; the excellent photostability and absence of blinking are very relevant for long-term observations [19,20] comparatively to fluorescent dyes and quantum dots; and, finally, their possible excitation in a large spectral range covering the first two NIR biological transparency windows [16,21] has led to several proof-of-concept studies in terms of in vivo imaging conditions [22], increased selectivity [23], acquisition time [24], and advanced theranostic applications [25,26].

Preparation of colloidal nanocrystal suspensions with size and morphology control to assess any possible scaling effects, and to further promote large-scale applications based on these multifunctional nanomaterials is thus very relevant; to do so, the so-called wet chemical routes are increasingly preferred [27]. Several chemical “recipes” leading to the reproducible preparation of NCO nanocrystals have thus been proposed [28], but future developments allowing a better size/shape control in wider ranges only rely on the detailed understanding and modification of the reaction pathway and growth mechanisms. Regarding LN, with, for instance, the Pechini method, use of inexpensive reagents such as citric acid, ethylene glycol, lithium carbonate, and ammonium oxalate niobate (V) hydrate results in crystallite sizes ranging from 20 nm to 230 nm (after a calcination step of the gel-like precursor between 500 °C and 800 °C), but with a high degree of aggregation [29]. Similarly, for the polymer approach, addition of poly(vinyl alcohol) and sucrose to an aqueous mixture containing hydrated ammonium oxalate-niobate and LiNO_3_ leads to very aggregated 36 nm nanocrystals after heating at 220 °C and calcination at 530 °C [30]. The traditional sol–gel route from Li and Nb alkoxides, already investigated in the 1980s, showed that dropwise addition of water only leads to the formation of a hydrated precipitate rich in ethoxy groups, but that further drying at 150 °C and annealing below 600 °C results in sub-20nm LN nanocrystals embedded in the amorphous organic matrix [31]. A sudden increase in the particle size to 300 nm is then observed after removal of the matrix above 600 °C. Later, a thermal treatment at 360 °C of a mix powder of lithium niobium isopropoxide and triphenylphosphine oxide as a capping agent has also been demonstrated to produce partially aggregated sub-10nm nanocrystals that rapidly evolve through oriented attachment into rod-like structures with lengths of up to 100 nm [32].

Use of mild hydrothermal/solvothermal conditions thus appears necessary, especially in the case of LiNbO_3_, as such conditions are well known to increase the solubility and reactivity of initial metal precursors under moderate temperatures and pressures, and, more importantly, to result in the formation of crystalline nanomaterials without subsequent high-temperature annealing treatment [33,34,35]. A mix of 3 µm long nanowires and sub-30 nm nanoparticles has thus been obtained from dry powders of niobium pentoxide and lithium hydroxide dissolved in water [36]. A combination of solvothermal conditions to produce first KNbO_3_ needles, and of a molten salt approach at 500 °C after mixing with LiNO_3_, successfully produced LN nanocubes but with a relatively large polydispersity with edge-length varying between 50 and 80 nm [37]. When alkoxide precursors were employed, Inui et al. [38] first reported the formation at 300 °C of flattened, crystallized particles of LN of average diameter 1 μm (with the so-called glycothermal method) in very good agreement with a more recent study [39]. Butanediol, niobium ethoxide and lithium acetate were then treated at 225 °C. Interestingly, under similar experimental conditions, smaller nanoparticles within the 20–50 nm size range were firstly obtained by Niederberger et al. with niobium ethoxide (Nb(OEt)_5_) also as the Nb source but with metal Li dissolved in benzyl alcohol [40]. For the chemical reaction pathway, formation of an oxo bridge after reaction between two alkoxide groups and ether elimination was suggested. The nanocrystal shape anisotropy was, on the other hand, not mentioned, although the experimental X-ray diffraction pattern evidenced reflections with different full width at half maximum (FWHM), in particular, the (110) and (006) peaks. Very similar results were also obtained from a commercial bimetallic precursor of lithium niobium ethoxide stabilized in its parent alcohol and after addition of different amounts of 1,4-butanediol as co-solvent [41]. More recently, niobium ethoxide dissolved in benzyl alcohol with lithium hydroxide and triethylamine as a surfactant also produced LN nanocrystals of tunable size between 30 and 95 nm. The reaction time was varied between 30 h and 96 h, thus supporting an Ostwald ripening in the last stages of the nanocrystal formation; however, for the earlier steps, the detailed chemical reactions leading to the oxide phase were not discussed [42]. Without surfactant and when the Li source was replaced with lithium acetylacetonate, solvolysis of the precursors by −OH groups was then proposed to account for the formation of sub-10 nm LN nanocrystals via condensation reactions [43].

In this article, special attention was paid to several experimental parameters that may affect the final nanocrystal size and shape when the bimetallic lithium niobium ethoxide stabilized in ethanol is solvothermally treated for 3 days at 230 °C. Addition of different amounts of co-solvent including butanol, 1,3-propanediol, 1,4-butanediol, and 1,5-pentanediol, as well as use of a methanolic commercial precursor, results in the formation of nanocrystals with different size and shape anisotropies. Demonstration of a simple ligand exchange upon addition of a glycol and thorough assessment of the nanocrystal size and shape distribution led us to propose a likely scenario for the reaction pathways and growth mechanisms. Thermal and chemical stabilities of the proposed monomer leading to the nucleation event together with the crystalline structure of LiNbO_3_, accounting for the formation of nanocrystals with adjustable mean size and shape anisotropy.

## 2. Materials and Methods

Preparation of LiNbO_3_ nanocrystals relies on a single step solvothermal process for 3 days at 230 °C in a 23 mL Teflon-lined stainless-steel autoclave (Parr-instrument). All chemicals of analytical grade were used as received without further purification. Alkoxide precursors were stored and handled under Argon unless specified in the text. A systematic variation of the experimental conditions (precursor concentration, presence of a co-solvent, and filling fractions of the autoclave) was achieved from variable amounts of lithium niobium ethoxide (5% w/v in ethanol, 0.135 M, Alfa Aesar, Thermo Fisher Scientific, Kandel, Germany) and the eventual addition of a co-solvent To investigate the influence of the latter, we specifically studied the effect of the chain length using of 1,2-ethanediol (E1,2), 1,3-propanediol (P1,3), 1,4-butanediol (B1,4), and 1,5-pentanediol (P1,5), while the influence of the number of hydroxyl groups was investigated with the help of butanol (B1), 1,4-butanediol (B1,4), and 1,2,4-butanetriol (B1,2,4). All co-solvents have been provided by Alfa Aesar except for 1,3-propanediol and 1,5-pentanediol purchased from abcr GmbH (Karlsruhe, Germany). A lithium niobium methoxide (5% w/v in methanol, Alfa Aesar) was also used to further demonstrate ligand exchange between the commercial precursors and 1,4-butanediol.

After cooling down to room temperature, nanocrystals were isolated from the reaction medium upon centrifugation (13,500 rpm) before being re-dispersed twice in ethanol for washing and additional centrifugation. Suspensions at a typical concentration of 1 mg/mL were then readily obtained in water or ethanol after sonication of the dried nanopowder with a standard sonic bath.

Structure analysis was carried out by X-ray diffraction (XRD) using a monochromatic Co-Kα_1_ radiation from an INEL CPS 120 instrument (INEL, Artenay, France), and the nanocrystal size along the different [hkl] directions was derived from the Scherrer’s formulae after careful calibration of the instrumental broadening with a LiNbO_3_ powder obtained from the grinding of a bulk crystal. Calculations of the apparent nanocrystal size S_hlk_ from the peak broadening of (hkl) reflections were performed by assuming a Lorentzian function for the peak shape. For some samples, this straightforward approach was also compared and was found to be very consistent with the S_hlk_ sizes calculated after extraction of the integrated intensities within FullProf according to the Le Bail global fitting procedure. Anisotropic broadening of the diffraction peaks (without contribution of the Gaussian part in the FWHM) and the absence of strain were assumed for the fitting procedure with a pseudo-Voigt function of the X-ray diffraction profiles acquired in this case with the Co-Kα_1_ and Co-Kα_2_ radiations of a PANalytical X’Pert^3^ Powder instrument (Malvern Panalytical, Palaiseau, France)equipped with a zero-background silicon sample holder.

Zeta-potential and dynamic light scattering (DLS) measurements (Zetasizer Nano, Malvern Panalytical, Palaiseau, France)) were used to assess the colloidal stability and aggregation state of the synthesized samples after re-dispersion in water or ethanol. Transmission electron microscopy (TEM) images (JEOL 2100 HT operating at 200 kV, JEOL Europe SAS, Croissy Sur Seine, France) were also recorded to further estimate the final nanocrystal size and shape polydispersity. Fourier Transform Infra-Red Spectrometry (FTIR) measurements in the attenuated-total-reflection configuration were performed from a Shimadzu (IRAffinity-1, Shimadzu France, Marne-la-Vallée) spectrophotometer.

## 3. Results

### 3.1. Synthesis without Co-Solvent

**Figure 1:**
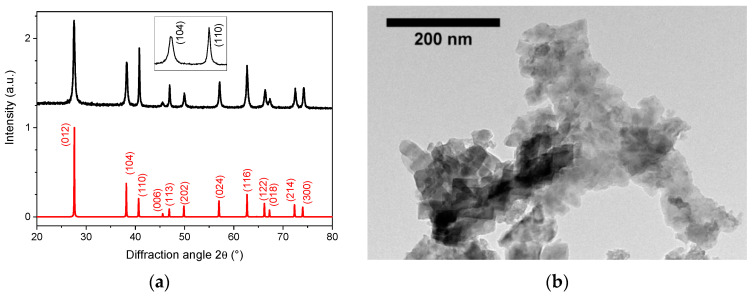
(**a**) XRD diffraction pattern of lithium niobate (LN) nanoplatelets after thermal decomposition of lithium niobium ethoxide in its parent alcohol. The reactant concentration was 0.135 M, and the different peak broadening is illustrated in inset for the (104) and (110) reflections. (**b**) Corresponding TEM image of LN nanoplatelets for which the anisotropic factor defined as S_110_/S_006_ was above 8.3.

Following the earlier works of Inui et al. [38] and Mohanty et al. [41], we achieved a systematic variation of the reaction conditions with the aim to better understand the reaction pathways and growth mechanisms possibly leading to LN nanocrystals with adjustable size and morphology. The synthesis temperature was fixed at 230 °C, namely, 20 °C below the maximum temperature that can be reached by the autoclave for a short period of time. As a reference synthesis, 5 mL of the commercial precursor solution corresponding to a volume fraction of 22% of the Teflon cup was first thermally treated for a period extending from 48 h to 72 h. After 3 days, the absence of a significant amorphous contribution was attested from the flat baseline observed in the corresponding XRD profile, showing formation of pure lithium niobate (Figure S1). In Figure 1, the X-ray diffraction pattern obtained after 3 days was further illustrated with a special emphasizes on the different FWHMs of the main (hkl) reflections. Deviations in terms of relative intensities and peak broadening were clearly visible after comparison with the reference pattern (ICSD # 80628). In addition, if a platelet morphology was also suggested from the corresponding TEM image, the shape anisotropy was more readily assessed from the apparent nanocrystal size S_hlk_ derived from the XRD data, which is summarized in Table 1 for the most intense (hkl) reflections.

**Figure A1:**
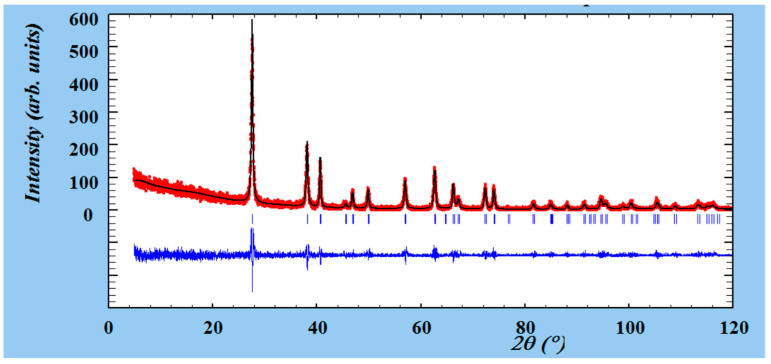
Le Bail profile refinement of the powder XRD pattern of LN nanocrystals obtained after thermal decomposition of a mixture containing 5 mL of lithium niobium ethoxide in its parent alcohol and 3.8 mL of 1,4-butanediol.

**Figure A2:**
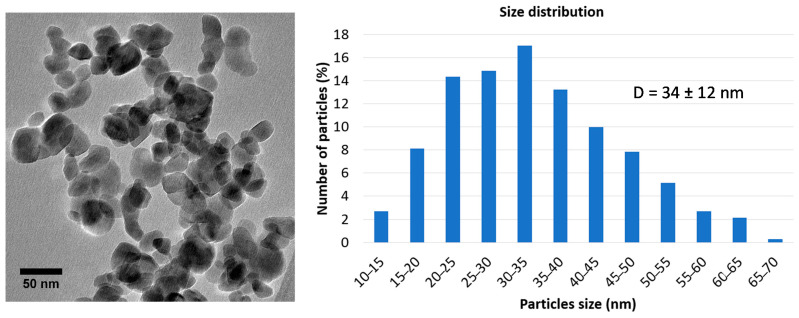
Representative TEM image of LN nanocrystals obtained after thermal decomposition of a mixture containing 5 mL of lithium niobium ethoxide in its parent alcohol and 3.8 mL of 1,4-butanediol. After considering 250 nanocrystals, we centered the corresponding histogram at 34 nm with a 12 nm standard deviation.

The lowest dimension at 12 nm was obtained along the c-axis direction from the (006) diffraction peak, whereas apparent sizes above 100 nm were derived perpendicularly from the (110) and (300) reflections. For these two diffractions peaks, the experimental FWHM was too close from the instrumental broadening to derive S_hlk_ values with a better accuracy. In the following, because the crystallite size calculated along each [hkl] direction was always located between the ones obtained from the (006) and (110) reflections, we introduced an anisotropic factor defined as *f* = S_110_/S_006_ in order to account for the particle anisotropy. According to Table 1, *f* was above 8.3 for the thermal decomposition of the commercial precursor without further additive. Note also that the addition of 3.8 mL of ethanol to the initial 5 mL of precursor volume decreased the reactant concentration from 0.135 M to 0.077 M but without any effect on the mean nanocrystal size defined along the [012] direction (see Appendix A and Figure A1 and Figure A2) and on the anisotropic factor as they were again derived at about 70 nm and above 8.0, respectively. The corresponding TEM image can be found in Figure S2.

These preliminary results obtained from the so-called reference synthesis differed a large amount from previously published results when 1,4-butanediol was added as a co-solvent [41] and in terms of size and pronounced shape anisotropy for LN nanocrystals obtained under solvothermal conditions [38,40]. As discussed in Section 4, we believe that such nanocrystal anisotropy is related to the polar non-perovskite crystalline structure of LiNbO_3_ and condensation reactions with different kinetics along and perpendicularly to the [001] direction.

### 3.2. Synthesis with Variable Amounts of 1,4-Butanediol Added as a Co-Solvent

Addition of a co-solvent for a given amount of precursor has several effects on the reaction medium. These include a change in the autogenous pressure, in the reactant concentration, and in the overall physicochemical properties of the reaction medium. For instance, change in the permittivity and viscosity of a co-solvent has indeed been shown to play a crucial role in the size and shape modifications of BaTiO_3_ nanoparticles [44,45]. Here, the filling fraction was first purposely changed since the autogenous pressure under solvothermal conditions is known to increase dramatically for a fixed temperature according to the molar volume of the solvent [46]. This physical parameter was investigated by increasing the volume fraction from 19% (2.5 mL of precursor and 1.9 mL of 1,4-butanediol) to 38% by doubling the initial amounts of precursor and co-solvent, thus keeping the same precursor concentration. The mean nanocrystal size S_012_ and anisotropic factor were estimated in both cases at 30 nm and 2.5, respectively, and the corresponding TEM images (Figure A2 and Figure S3) also showed no visible differences, suggesting a negligible effect of the autogenous pressure, contrary to what was previously suggested [41].

**Figure 2:**
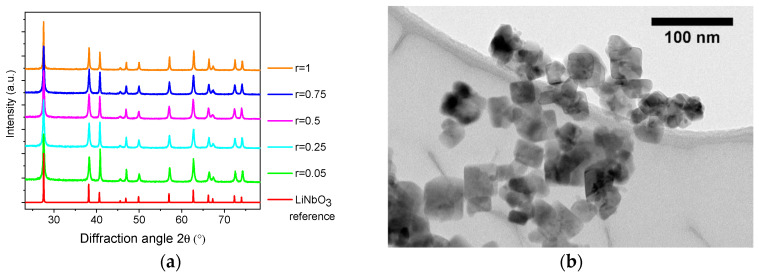

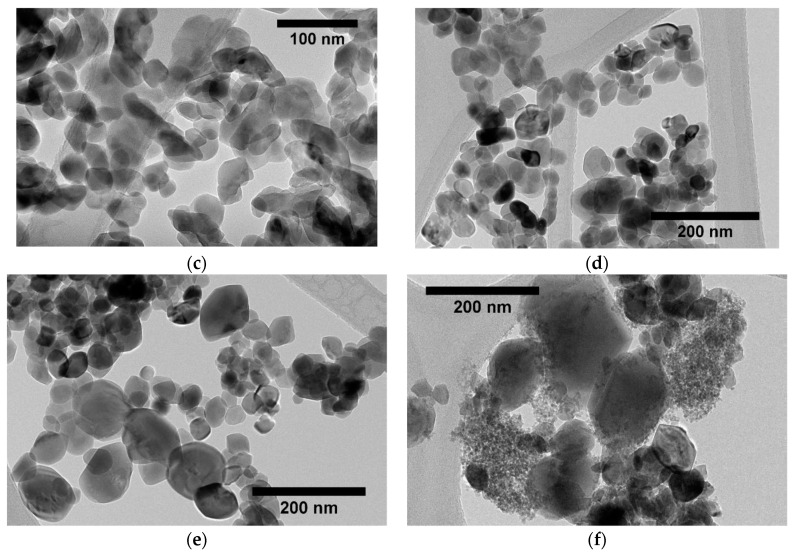
(**a**) XRD diffraction patterns of LN nanocrystals for increasing values of the molar ratio *r* of 1,4-butanediol to ethanol. Corresponding TEM images for (**b**) *r* = 0.05, (**c**) *r* = 0.25, (**d**) *r* = 0.5, (**e**) *r* = 0.75, and (**f**) *r* = 1.

The reactant concentration can also be varied according to the molar ratio *r* defined here as the molar concentration of 1,4-butanediol to ethanol in the precursor. For a volume of precursor fixed at 5 mL, the addition of 0.38 mL (*r* = 0.05), 1.9 mL (*r* = 0.25), 3.8 mL (*r* = 0.5), 5.7 mL (*r* = 0.75), and 7.6mL (*r* = 1.0) of 1,4-butanediol was found to strongly influence the mean nanocrystal size and anisotropic ratio after treatment of the XRD data (see Table 2). Note that the corresponding XRD patterns (Figure 2a) demonstrated the absence of any phase impurity.

If a substantial decrease in the corresponding S_012_ values was observed below *r* = 0.5, together with a significant reduction of the shape anisotropy comparatively to the reference synthesis, we found that TEM imaging (Figure 2) also revealed that the size polydispersity was severely affected for *r* > 0.5. Monocrystalline nanoparticles of very different sizes were clearly observed, indicating, as discussed in Appendix A, that the mean nanocrystal size derived from the XRD data must be considered with caution for very polydisperse samples. Interestingly, the size increase at high 1,4-butanediol content was consistent with the formation of micrometer disk-shaped particles produced when lithium acetate and niobium ethoxide are solvothermally treated in pure 1,4-butanediol [38,39], indicating a low nucleation rate in that case. On the contrary, nanocrystal growth was less predominant for the *r* values fixed between 0.05 and 0.5. The mean nanocrystal size calculated at about 30 nm was then in good agreement with the TEM images for these samples showing the best characteristics in terms of size and shape anisotropy.

For the molar ratio at *r* = 0.5, a possible artifact arising from the condition storage is mentioned here. Regarding the increase in the size and shape polydispersity, we indeed noticed that successive openings and storage of the precursor solution under ambient conditions had a huge impact on the nanocrystal morphology, as demonstrated in Figure S4. After ageing times of 1 month and 3 months, the size and shape polydispersity was not only increased but facetization was also more pronounced. Alkoxide precursors are indeed known for their sensitivity to moisture [47,48,49], such that even tiny amounts of water can interfere with the reaction pathways and growth mechanisms discussed in Section 4.

### 3.3. Synthesis with Different Glycols Added as a Co-Solvent

Ligand exchanges for niobium and other alkoxides are also well-established [50,51], and thus influence of the co-solvent on the nanoparticle size and shape was also investigated by addition of 1,2-ethanediol, 1,3-propanediol, 1,4-butanediol, or 1,5-pentanediol to the precursor at a molar ratio of glycol to ethanol fixed at 0.5. As demonstrated in Figure S5, phase-pure LN nanocrystals were obtained for the last three glycols. Estimation of the nanocrystal size along the [012], [110], and [006] directions and anisotropic ratio are reported in Table 3 for all the co-solvents, with the exception of 1,2-ethanediol for which the peak broadening of all reflections was very close to the instrumental resolution and because of the presence of a phase impurity. Note also that colloidal stability could not be achieved for this sample. For the others, a continuous decrease of the mean nanocrystal size S_012_ according to the glycol chain length was clearly observed along with a significant reduction of the anisotropic ratio comparatively to the reference syntheses.

**Figure 3:**
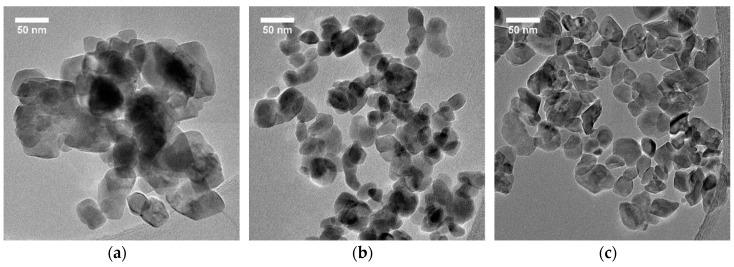
TEM images of LiNbO_3_ nanocrystals showing the influence of the glycol chain length for a molar ratio of glycol to ethanol at *r* = 0.5. For a precursor volume fixed at 5 mL, we added (**a**) 3.1 mL of 1,3-propanediol, (**b**) 3.8 mL of 1,4-butanediol, and (**c**) 4.5 mL of 1,5-pentanediol.

Interestingly, calculation of the nanocrystal sizes from XRD data were found to be in good agreement with the TEM images, showing, as illustrated in Figure 3, a decrease of the particle size as long as the glycol chain length was increased.

In agreement with the S_hkl_ values derived in Table 3, samples prepared with 1,4-butanediol and 1,5-pentanediol were found to be the most monodisperse since a clear reduction of the size and shape distribution was observable. Deviation from the ideal spherical shape was still noticeable, however. After dispersion in ethanol or water, an excellent colloidal stability was also achieved for the samples of Figure 3 with typical zeta potential values measured at about −50 mV and DLS size distributions in number decreasing from ≈130 nm for 1,3-propanediol to ≈80 nm for 1,4-butanediol and 1,5-pentanediol.

With the aim to better understand the influence of the co-solvent in terms of reaction pathways, growth mechanisms, and colloidal stability, we changed the number of hydroxyl groups with the use of butanol and 1,2,4-butanetriol. If well-crystallized LN samples could not be produced with that latter highly viscous co-solvent, we consider the monohydroxy alcohol of special interest since only simple ligand exchange can occur without formation of chelates between two niobium alkoxides. Such a scenario has been evidenced, for instance, in the solvothermal production of metal chalcogenides with ethylenediamine as the solvent [52]. Here, 1,4-butanediol has indeed two hydroxyl groups possibly leading to the formation of such chelates. Similarly, cyclic ethers such as tetrahydrofuran arising from intramolecular condensation [53] cannot be released during the thermal treatment as already evidenced for the glycothermal preparation of ZrO_2_ from several Zr alkoxides [54]. To keep the molar ratio at *r* = 0.5, we mixed 4 mL of butanol with 5 mL of the precursor solution, resulting in very similar results to the ones obtained with 1,4-butanediol, namely, a S_012_ value at 21 nm for an anisotropic ratio of 2.8. The TEM image provided in Figure S6 also evidences very comparable morphologies for the two co-solvents, although colloidal stability could not be achieved for nanocrystals prepared with butanol.

## 4. Discussion

Comparatively to the pioneering work of Inui et al. [38], who obtained flattened LN particles in the micrometer range in pure 1,4-butanediol, and the more recent one of Mohanty et al. [41], who produced more or less faceted LN nanocrystals of nearly 50 nm size with variable amounts of 1,4-butanediol added as a co-solvent to an ethanolic solution of mixed lithium niobium ethoxide, understanding the role of such glycol under solvothermal conditions is aimed here to prepare LN nanocrystals with adjustable size and morphology. Starting from reference syntheses without any co-solvent, we obtained nanocrystals with a mean size at about 70 nm and a pronounced platelet-like morphology, as illustrated from the high values of the defined anisotropic ratio *f* = S_110_/S_006_. Addition of variable amounts of 1,4-butanediol corresponding to molar ratios comprising between 0.05 and 0.5 not only results in the strong decrease of the S_012_ size from 70 nm to ≈30 nm and of *f* from 8.3 to ≈2.6, but also to an excellent colloidal stability. Samples with mass concentration below 0.1 mg/mL showed no sign of sedimentation after several months when dispersed in ethanol. According to FTIR measurements, this stability can be partially attributed to the residual presence of glycoxy chains whose terminal hydroxyl groups promote higher electrostatic repulsion comparatively to the synthesis done with butanol or with ethanol only (Figure S7). Interestingly, nature of the alkoxy groups is also known to strongly affect the nucleation/growth kinetics and the final nanoparticle morphology [55]. Here, the absence of chelates between two metal Nb and a simple ligand exchange between the primary ethoxy groups and the different glycols were demonstrated from the very similar results obtained with butanol and 1,4-butanediol for 0.05 < *r* < 0.5. This is also supported by the data obtained when the ethoxide precursor was replaced by a lithium niobium methoxide in its parent alcohol. The mean nanocrystal size was indeed derived at 33 nm for an anisotropic ratio at 2.5 when 4.9 mL of 1,4-butanediol was added to 5 mL of the precursor solution to keep a molar ratio at *r* = 0.5. XRD measurements and TEM imaging (Figure S8) also revealed a nanocrystal morphology very similar to the one observed with the ethoxide precursor mixed with 1,4-butanediol or butanol at *r* = 0.5 (Figure S6).

To further elucidate the effect of the co-solvent and ligand exchange on the final nanocrystal morphology, we here discuss the reaction pathway and growth mechanism leading to the mixed metal-oxide network. Addition of water to the reaction medium or heating for the so-called non aqueous sol–gel synthesis are the two well-established routes to promote condensation and crystallization when alkoxides are used as precursors [47,55]. If water was not purposely added here, a hydrolytic reaction pathway can occur though if water is produced in situ through esterification reactions or alkene formation [56,57]. FTIR analysis of the reaction medium at the end of the solvothermal treatment (Figure S9) was thus performed and compared with the one of pure ethanol and with the one of water. The characteristic absorption band of water at 1660 cm^−1^ could not be detected in the reaction medium as a by-product, even if one cannot expect more than 10^−3^ mol of water (18 µL) according to the initial amount of alkoxide groups. Note that such a low volume of water was still above the detection limit of our FTIR setup as it could be detected after mixing it with 5 mL of absolute ethanol. Because of the non-aqueous solvothermal conditions here applied, thermally induced aprotic condensation reactions are thus to be considered [53,58]. According to the available literature data, the reaction pathway we suggest is very likely the following.

After mixing the commercial precursor solution with a glycol, we can write a room-temperature ligand exchange reaction [50,51] as follows:

(1) $$\mathrm{LiNb}{\left(\mathrm{OR}\right)}_{6}+6\;R\prime \mathrm{OH}⇆\mathrm{LiNb}{\left(\mathrm{OR}\prime \right)}_{6}+6\;\mathrm{ROH}$$

where R stands for −C_2_H_5_ and R′ for −C_4_H_8_OH when 1,4-butanediol is used as a co-solvent. Upon heating, a thermal dissociation of the modified mixed precursor is expected, as originally demonstrated by Mehrotra et al. for several niobium (and tantalum) alkoxides [59]:

(2) $$\mathrm{LiNb}{\left(\mathrm{OR}\prime \right)}_{6}⇆\mathrm{Nb}{\left(\mathrm{OR}\prime \right)}_{5}+{\;\mathrm{Li}}^{+}+{\;R\prime O}^{-}$$

Further heating leads to the thermal decomposition of the Nb alkoxide complexes and results in the formation of ethers as initially postulated by Bradley et al. [60] and later evidenced from gas chromatography for the formation of LiNbO_3_ microparticles [38]. We mention here that the produced ether could not be detected from FTIR measurements possibly because of its expected high volatility and the similar characteristic vibration bands at 1065 cm^−1^ and 1045 cm^−1^ for the CO bond of ethers and ethanol, respectively. The large content of ethanol and the absence of characteristic IR bands of the expected ether out of the main vibrations of the solvent and co-solvent thus require use of other spectroscopic techniques. Moreover, we point out that thermal decomposition of Nb alkoxide [50,60] results in the formation of a Nb = O double bond and thus in a negatively charged oxoalkoxide.

$$\mathrm{Nb}{\left(\mathrm{OR}\prime \right)}_{5}⇆{\left[\mathrm{NbO}{\left(\mathrm{OR}\prime \right)}_{4}\right]}^{-}+{\;R\prime }^{+}$$

(3) $${R\prime }^{+}+{\;R\prime O}^{-}⇆{\;R\prime }_{2}O$$

The positively charged carbocations ${R\prime }^{+}$ of varying lengths according to the co-solvent used can then react with the ethoxide groups produced from reaction (2). The oxoalkoxide species also combine with Li^+^ ions to produce a zero-charged precursor or monomer, a necessary condition before condensation and nucleation can take place [47,61]:

(4) $${\left[\mathrm{NbO}{\left(\mathrm{OR}\prime \right)}_{4}\right]}^{-}+{\;\mathrm{Li}}^{+}⇆{\;\mathrm{Li}}^{+-}[\mathrm{NbO}{\left(\mathrm{OR}\prime \right)}_{4}]$$

**Figure 4:**
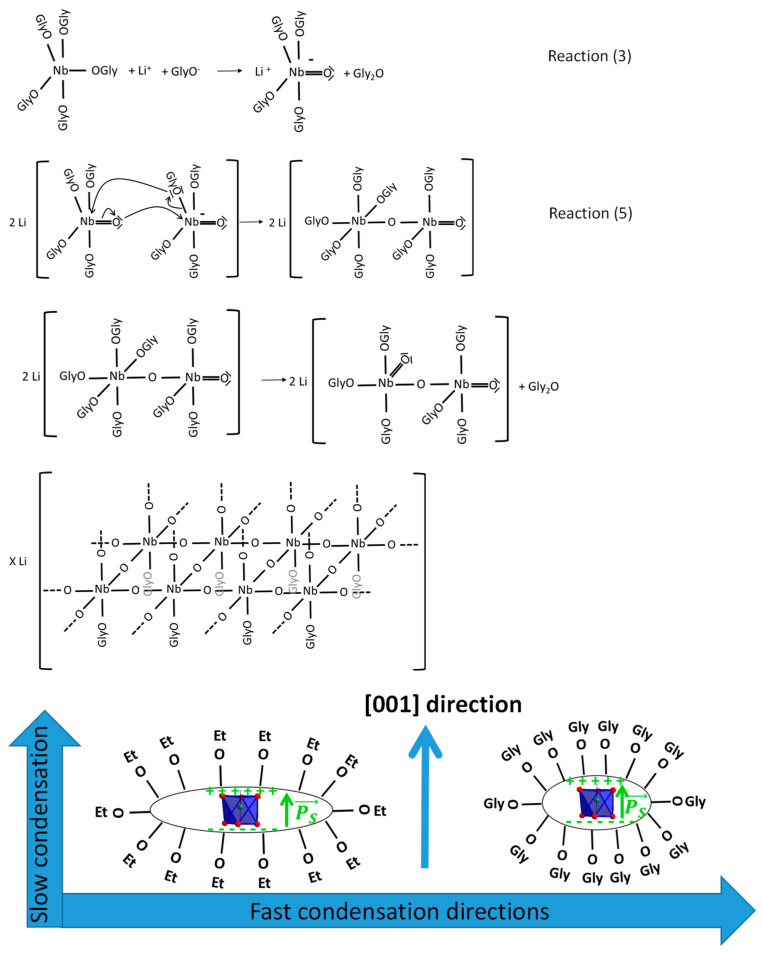
Scheme of the reaction pathway leading to LiNbO_3_ nanocrystals with different shape anisotropy. GlyO and EtO are glycoxy and ethoxy groups corresponding to −OC_4_H_8_OH and −OC_2_H_5_, respectively. The reactions (3) and (5) and the subsequent condensation reactions are described in the text. An oxygen octahedra (in blue) surrounding Nb^5+^ ions is also illustrated in the lower panel together with the spontaneous polarization Ps (green arrow) pointing towards the [001] direction.

As illustrated in Figure 4, nucleophile attack of Nb centers by one of the lone pair of oxygen then leads to condensation through inorganic polymerization reactions and to the formation of primary clusters and nuclei.

(5) $$2{\;\mathrm{Li}}^{\pm }[\mathrm{NbO}{\left(\mathrm{OR}\prime \right)}_{4}]⇆{\mathrm{Li}}^{\pm }[\mathrm{Nb}{\left(\mathrm{OR}\prime \right)}_{4}]―O―{\left[{\left(\mathrm{OR}\prime \right)}_{3}\mathrm{ONb}\right]}^{\mp }\mathrm{Li}+{\;R\prime O}^{-}\;⇆{\mathrm{Li}}^{+-}[\mathrm{Nb}{\left(\mathrm{OR}\prime \right)}_{5}]―O―{\left[{\left(\mathrm{OR}\prime \right)}_{3}\mathrm{ONb}\right]}^{-+}\mathrm{Li}$$

After the formation of the first Nb―O―Nb bond, elimination of the five remaining $R^{\prime }$ groups proceeds similarly to reaction (3). A positively charged carbocation is first released, leading to the formation of a new Nb = O double bond. This is accompanied by the internal elimination of a glycoxy group ${R\prime O}^{-}$ that reacts with the leaving $R^{\prime +}$ group to produce the corresponding ether (Figure 4). Condensation through addition of new monomers to the cluster and nuclei then continues with the nucleophile attack of Nb centers by one of the lone pair of a monomer, thus keeping Nb ions octahedrally coordinated through the successive elimination of R′ groups. As discussed below, growth then proceeds after addition of new monomers to the glycoxy-stabilized nuclei and primary nanocrystals.

The later stages of the growth mechanisms to obtain uniform nanocrystals has been intensively discussed in the literature and assumes a burst of nucleation and a diffusion-controlled growth by addition of monomers to the growing particles [28,62,63]. Such a separation between the nucleation and growth processes thus relies on a short nucleation step, implying a high concentration of monomers to reach the required high supersaturation level for homogeneous nucleation. Afterwards, their rapid consumption not only quickly lowers the supersaturation level but also limits the number of available monomers for the subsequent growth. For the heat-up method we used here, size uniformity should thus be controlled by the thermal decomposition of reaction (3) since the latter determines the rate of monomer formation, leading to a zero-charged precursor. A higher thermal stability of the $\mathrm{Nb}{\left(\mathrm{OR}\prime \right)}_{5}$ species can first be assumed as long as the length of the R′ chain is increased [60]. In agreement with the partial charge model [47], a better electronic stabilization of the metal centers by electro-donating groups is indeed obtained after the initial ligand exchange with glycoxy groups of varying chain lengths made of three, four, or five carbons in our case (inductive effect). Concomitantly, the thermal decomposition reaction (3) produces ${R^{\prime }}^{+}$ carbocations as reaction intermediates. Stability of these carbocations, and more precisely of the positively charged carbon, requires electro-donating groups such as −CH_2_, the number of which is four and five in the case of 1,4-butanediol and 1,5-pentanediol, respectively. Although the glycoxy ligands end with an electro-attracting hydroxyl group, influence of this latter on the positively charged carbon is limited since inductive effects are not significant for a distance above three chemical groups [64]. The higher thermal stability of the $\mathrm{Nb}{\left(\mathrm{OR}\prime \right)}_{5}$ species together with the increased stability of ${R\prime }^{+}$ carbocations with long carbon chains thus promote the rapid formation of reactive monomers and smaller nanocrystals in agreement with the decrease of the mean nanocrystal size we derived in Table 2 and Table 3 for *r* values located between 0.05 and 0.5. On the contrary, when *r* was fixed above 0.75, a reduced nucleation rate arising for the higher co-solvent viscosity and a prolonged period of nanocrystal growth accounted for the formation of strongly size-polydisperse samples. Compared with the reference synthesis, the above arguments are also consistent with the size reduction already measured when 1,3-propanediol was added as a co-solvent and the preparation of larger particles above 100 nm when 1,2-ethanediol was used. Finally, we also point out in our case a weak effect of the dielectric constant on the stability of the carbocations and overall nucleation step. If a high relative permittivity promotes the preparation of smaller ZnO nanocrystals from the solvothermal decomposition of zinc acetate in various glycols ranging from 1,3-propanediol (ε_r_ = 35.10) to 1,5-pentanediol (ε_r_ = 26.10) [65], weakening of the electrostatic interactions has almost no effect here since larger nanocrystals are obtained with the co-solvent of higher dielectric constant.

Regarding the shape anisotropy already noticeable from the XRD data in the reference work of Niederberger et al. [40] and in other more recent papers after careful examination of the published XRD patterns [41,42], one can first point out that several perovskite nanocrystals, belonging to more isotropic cubic or pseudo-cubic lattice symmetries, and prepared from alkoxide precursors under controlled addition of water, do not display such an anisotropic behavior [12,45,66,67]. A key difference between the perovskite crystalline structure of BaTiO_3_ (or its derived ternary, quaternary, and quinary oxides after isovalent chemical substitution of Ba^2+^ and Ti^4+^ ions) and the crystalline structure of LiNbO_3_ belonging to the 3m point group is a drastic change in the arrangement of the oxygen octahedra surrounding the Nb^5+^ and Li^+^ cations. As illustrated in Figure S10, the distribution of Nb^5+^ ions within the crystalline structure can be described from oxygen corner-sharing octahedra and there was a similar arrangement around Li^+^ ions. The arrangement of two adjacent oxygen octahedra surrounding Nb^5+^ and Li^+^ was, however, very different as they exhibited edge-sharing octahedra perpendicularly to the *c*-axis direction and face-sharing octahedra along the polar direction. Because of the strong electrostatic repulsions between Li^+^ and Nb^5+^ cations whose separations along and perpendicularly to the *c*-axis were 3.0425 Å and 3.0608 Å, respectively, face-sharing octahedra along the polar direction were less favored [68], and this contributed to slower condensation reactions during the nucleation and initial stages of the growing steps along the *c*-axis. For the reference synthesis without co-solvent, nanocrystals were indeed obtained with the highest anisotropic ratio *f* measured above 8. When a glycol was added to the reaction medium, the anisotropic ratio was already strongly reduced for a *r* value of 0.05 corresponding to an equal mole number of initial ethoxy groups and 1,4-butanediol molecules (Table 2). Regarding the growth mechanism after the complete ligand exchange supported here by the similar results obtained for the *r* values located between 0.05 and 0.5, a controlled agglomeration of primary nuclei followed by an Ostwald ripening process would be unlikely, contrary to what was observed with the hydrolysis of niobium ethoxide after mixing with lithium hydroxide monohydrate in the surfactant assisted solvothermal route developed by Gates et al. [42]. Time-dependent measurements after 12 h and 24 h did not indicate in our case a non-uniform growth of the nanocrystals along the different [hkl] directions (data not shown). A uniform decrease of the peak broadening was indeed monitored, corresponding to an almost constant size increase at about 10 nm for all the main (hkl) reflections. Addition of new monomers to the primary nuclei and growing nanocrystals was thus assumed here.

However, the anisotropic ratio was found to vary from 8.3 to ≈2.6 after the addition of different glycols to the precursor solution, and thus the presence of face-sharing octahedra along the polar direction was not the only contribution to the observed variable shape anisotropy. As depicted in Figure 4, growing of the primary nuclei by addition of new monomers was also accompanied by the build-up of a polar order, or spontaneous polarization, along the *c*-axis once crystallization occurred. We thus assumed that Nb atoms belonging to the negatively-charged (00-1) plane of the {001} facet were less prone to the nucleophile attack of new monomers comparatively to the other [hkl] growth directions. The surface glycoxy groups of the (00-1) plane were, however, more readily removed than the ethoxy groups corresponding to the reference synthesis, as measured from the decrease of the anisotropic ratio. Long carbon-chain glycoxy groups are supposed to also have a better screening effect against the polarization, thus reducing the difference in the growth velocities observed parallel and perpendicularly to the *c*-axis direction.

## 5. Conclusions

Synthesis of phase-pure, highly crystalline sub-50 nm LiNbO_3_ nanocrystals with adjustable mean size and shape anisotropy was demonstrated from the non-aqueous sol–gel route. Mixed metal alkoxide precursors were treated for 3 days under standard solvothermal conditions upon addition of different glycols as co-solvent, leading to a simple ligand exchange with primary ethoxy/methoxy groups of both ethanolic and methanolic precursors, respectively. According to the carbon chain length of the tested glycols, the mean nanocrystal size derived along the [012] direction could be reduced from 47 nm to 27 nm along with a drastic reduction of the shape anisotropy comparatively to the simple thermal decomposition of the precursor alone. The most monodisperse samples obtained with 1,4-butanediol and 1,5-pentanediol were still evidenced though a platelet-like morphology with an anisotropic ratio at about 2.5. However, because of their colloidal stability and reduced size and shape distributions, such samples can be implemented as new calibration probes for second harmonic spectroscopic studies over broad excitation ranges thanks to the absence of size and shape effects in the orientation-averaged second-order susceptibility of sub-50 nm LN nanocrystals and to their inherent non-resonant SHG response [17,21]. After surface functionalization, advanced theranostic applications based on the SH properties of LN nanocrystals have also been recently demonstrated for the controlled and local release of the anticancer drug chlorambucil [69]. Noteworthy, in this study, the systematic variation of the reaction medium composition allowed us to suggest a reaction pathway on the basis of the available literature data addressing the thermal stability of niobium alkoxides. Thermal stability of the $\mathrm{Nb}{\left(\mathrm{OR}\prime \right)}_{5}$ species and formation of positively charged carbocations accounted for the quicker formation rate of monomers and smaller nanocrystal size when long carbon chain glycols were used and for *r* values in the 0.05–0.5 range. Regarding the residual shape anisotropy, the non-perovskite crystalline structure of LiNbO_3_ seemed to play a key role during the nanocrystal growth. Contrary to recently published studies on various perovskite nanocrystals of the BaTiO_3_ family, the unfavorable arrangement of face-sharing oxygen octahedra along the polar direction of LiNbO_3_ was first suggested as a slower growth velocity was observed along [001]. An interplay between the presence of different surface ethoxy/glycoxy groups on the (00-1) plane and the build-up of a polar order as crystallization occurred was also assumed to account for the variable shape anisotropy.

Finally, we also want to point out that our preliminary studies indicated similar reaction pathways and growth mechanisms when the mixed metal precursors were replaced by a simple mixing of the cheaper precursors made of pure niobium ethoxide and lithium ethoxide. On the basis of our experience obtained with several commercial mixed metal precursors coming from different batches and manufacturers, we also advise assessing purity of the parent alcohol. In a few cases, an excess of toluene instead of ethanol, for instance, was found thus resulting in the reproducible formation of non-phase pure LiNbO_3_ nanocrystals.

## Figures and Tables

**Table 1:** Apparent nanocrystal sizes, S_hlk_ (in nm), from the Scherrer’s formulae for LN nanoplatelets after thermal decomposition of lithium niobium ethoxide in its parent alcohol for 3 days at 230 °C.

S_012_	S_014_	S_110_	S_006_	S_113_	S_202_	S_024_	S_116_	S_112_	S_018_	S_214_	S_300_
69	52	>100	12	98	>100	78	59	>100	31	>100	>100

**Table 2:** Evolution of the mean nanocrystal size S_012_ (in nm) and anisotropic ratio for a fixed volume of precursor at 5 mL and increasing values of *r* upon addition of 1,4-butanediol. The number of moles of ethanol was 8.57 × 10^−2^, whereas for 1,4-butanediol it was increased from 4.30 × 10^−3^ (*r* = 0.05) to 8.57 × 10^−2^ (*r* = 1.0). The number of moles of ethoxy groups coming from the alkoxide precursor was 4 × 10^−3^ in each case.

Composition ^1^	*r*	S_012_ (nm)	S_110_/S_006_
5 mL of P	0	69	>8.3
5 mL of P (48 h)	0	49	>8.3
5 mL of P + 0.38 mL of B1,4	0.05	29	4.9
5 mL of P + 1.9 mL of B1,4	0.25	32	3
5 mL of P + 3.8 mL of B1,4	0.5	33	2.4
5 mL of P + 5.7 mL of B1,4	0.75	45	2.6
5 mL of P + 7.6 mL of B1,4	1	91	2.9

^1^ The commercial precursor is denoted as P and the reaction time at 230 °C is specified when it differed from 72 h.

**Table 3:** Evolution of the nanocrystal sizes S_012_, S_110_, and S_006_ (in nm) and anisotropic ratio for a fixed volume of precursor at 5mL and various co-solvents of increasing chain lengths. The molar ratio of glycol to ethanol was 0.5 in each case.

Composition ^1^	S_012_ (nm)	S_110_ (nm)	S_006_ (nm)	S_110_/S_006_
5 mL of P + 3.1 mL of P1,3	47	58	20	2.9
5 mL of P + 3.8 mL of B1,4	30	42	16	2.6
5 mL of P + 4.5 mL of P1,5	27	52	20	2.6

^1^ The commercial precursor is denoted as P.

## Data Availability

The data presented in the study can be requested from the authors.

## Supplementary Materials

The following are available online at https://www.mdpi.com/2079-4991/11/1/154/s1: Figure S1: XRD diffraction patterns of LN nanopowders obtained after a thermal treatment at 230 °C of the commercial precursor alone for a period extending from 48 h to 72 h. After 3 days, the absence of a significant amorphous contribution was attested from the almost flat baseline on the corresponding XRD profile. Figure S2: TEM image of LN nanoplatelets after dilution with ethanol of the precursor solution giving a molar concentration fixed at 0.077 M. Data treatment of the XRD profile (data not shown) resulted in a mean nanocrystal of S_012_ = 77 nm and an anisotropic factor f > 8.0. Figure S3: TEM images of LN nanocrystals at different filling fractions of the Teflon cup. (left) We used 2.5 mL of precursor and 1.9 mL of 1,4-butanediol for a filling fraction at 19% and (right) 5 mL of precursor and 3.8 mL of 1,4-butanediol for a filling fraction at 38%. TEM images are very similar, with the mean nanocrystal size S_012_ and anisotropic factor estimated at 30 nm and 2.5, respectively, in both cases. Effect of the autogenous pressure was negligible. Figure S4: Influence of the ageing time on a precursor kept under ambient conditions for a composition of the reactive medium corresponding to 5 mL of precursor and 3.8 mL of 1,4-butanediol. TEM images of LiNbO_3_ nanocrystals for a synthesis performed with a freshly new precursor (left) and after a few openings of the same precursor solution kept under ambient conditions for an ageing time of 1 month (middle) and 3 months (right). Note how the size and shape polydispersity was strongly affected in terms of facetization with the appearance of flattened cubic-shape nanocrystals when the precursor solution was not handled under an inert atmosphere. Figure S5: (Left) XRD diffraction patterns of LN nanopowders obtained after a thermal treatment at 230 °C for 3 days of the commercial precursor with various co-solvents of increasing chain lengths. The molar ratio of glycol to ethanol was 0.5 in each case. (right) Closer view at 2θ ≈27.5° of the (012) reflection showing a larger FWHM as long as the glycol chain was increased. Figure S6: Comparison of the TEM images for LiNbO_3_ nanocrystals produced at a molar ratio of 0.5 when butanol (left) and 1,4-butanediol (right) were added to 5 mL of the ethanolic precursor solution. Structure of each co-solvent is indicated in the upper panel and the corresponding optical images, illustrating the absence of colloidal stability for the LiNbO_3_ nanocrystals prepared with butanol and dispersed at 0.1 mg/mL in ethanol after a period of 5 days. Figure S7: Comparison of FTIR spectra in the 2500–4000 cm^−1^ spectral range for LiNbO_3_ nanocrystals obtained with and without 1,4-butanediol. With the glycol, the higher amount of hydroxyl groups and aliphatic CH groups is visible from the large band at 3500 cm^−1^ and the two peaks below 3000 cm^−1^, respectively. Figure S8: (left) XRD diffraction pattern of LN nanocrystals obtained after a thermal treatment at 230 °C for 3 days of 5 mL of lithium niobium methoxide dissolved in methanol after addition of 4.9 mL of 1,4-butanediol. (right) Corresponding TEM image showing a nanocrystal morphology very similar to the one observed in Figure S6. Figure S9: Comparison of the FTIR spectra of water and ethanol with that of the reaction medium at the end of the 3-day solvothermal treatment did not evidence the characteristic absorption band of water at 1660 cm^−1^. Figure S10: (a) Arrangement of the edge-sharing octahedra surrounding the Nb^5+^ (in blue) and the Li^+^ ions (in yellow) in the (002) crystalline plane. (b) Partial view of the face-sharing octahedra surrounding Nb^5+^ and Li^+^ along the polar direction whereas an isotropic corner-sharing octahedra arrangement was visible if only Li^+^ (or Nb^5+^) ions were considered.

## Appendix A

Assessment of the mean nanocrystal size and shape anisotropy from TEM observations and data treatment of the XRD profiles is detailed here from a sample obtained after mixing of 5 mL of the precursor with 3.8 mL of 1,4-butanediol. Such samples are among the most monodisperse in size and shape but still evidence a size and shape distribution that complicates the exact derivation of the nanocrystal morphology. The Le Bail profile refinement of the powder XRD pattern leading to a residual χ^2^ value at 1.77 is depicted in Figure A1, from which the derived crystallite sizes along the [012], [110], and [006] directions were estimated at 30 nm, 43 nm, and 17 nm, respectively. Note that these values were in complete agreement with those of Table 3 obtained from the Sherrer’s formulae. The corresponding anisotropic factor was then 2.6 and, very interestingly, the refined cell parameters (a = 5.1474 (2) Å, c = 13.8789 (1) Å) were very close to the ones obtained by Kimura et al. [70] for stoichiometric lithium niobate crystals.

These values are to be compared with the size distribution here obtained after considering 250 nanocrystals on the corresponding TEM images. Because of the platelet morphology, partial overlapping of several individual nanocrystals, possible various inclinations on the TEM grid, and a given size distribution along each [hkl] direction, we obtained a relative broad size distribution centered at 34 nm. This latter value is, however, in relative good agreement with the nanocrystal size calculated at 30 nm from the (012) reflection. In the paper overall, we thus considered S_012_ as a mean nanocrystal size for a better comparison of the different samples prepared under different experimental conditions. Importantly, if a mean nanocrystal size and standard deviation can be readily obtained from TEM imaging, the mean nanocrystal along a specific [hkl] direction is much more difficult to estimate in that case. Finally, for further comparison with other experimental techniques, the mean DLS size for this sample was measured at ≈80 nm from the size distribution in number and was indicative of the small number of agglomerates in water- or ethanol-based colloidal suspensions.
