# Supplementary material for: On the Reaction Pathways and Growth Mechanisms of LiNbO3 Nanocrystals from the Non-Aqueous Solvothermal Alkoxide Route
Source: Nanomaterials (Basel). 2021 Jan 9;11(1):154. doi: 10.3390/nano11010154 (PMC7828100; doi:10.3390/nano11010154)
Supplement: Supplementary file 1 [file nanomaterials-11-00154-s001.pdf]

Supplementary Materials

On the Reaction Pathways and Growth Mechanisms of $LiNbO_3$ Nanocrystals from the Non-Aqueous Solvothermal Alkoxide Route

Mathias Urbain [1], Florian Riporto [1], Sandrine Beauquis [1], Virginie Monnier [2], Jean-Christophe Marty [1], Christine Galez [1], Christiane Durand [1], Yann Chevolot [2], Ronan Le Dantec [1] and Yannick Mugnier [1,*]

[1] Univ. Savoie Mont Blanc, SYMME, F-74000 Annecy, France; mathias.urbain45@gmail.com (M.U.); florian.riporto@gmail.com (F.R.); Sandrine.Beauquis@univ-smb.fr (S.B.); Jean-Christophe.Marty@univ-smb.fr (J-C.M.); Christine.Galez@univ-smb.fr (C.G.); Christiane.Durand@univ-smb.fr (C.D.); Ronan.le-Dantec@univ-smb.fr (R.L.D.)

[2] Université de Lyon, Ecole Centrale de Lyon, UMR CNRS 5270, Institut des Nanotechnologies de Lyon (INL), F-69134 Ecully Cedex, France;, Virginie.Monnier@ec-lyon.fr (V.M.); Yann.Chevolot@ec-lyon.fr (Y.C.)

* Correspondence: Yannick.Mugnier@univ-smb.fr (Y.M.)

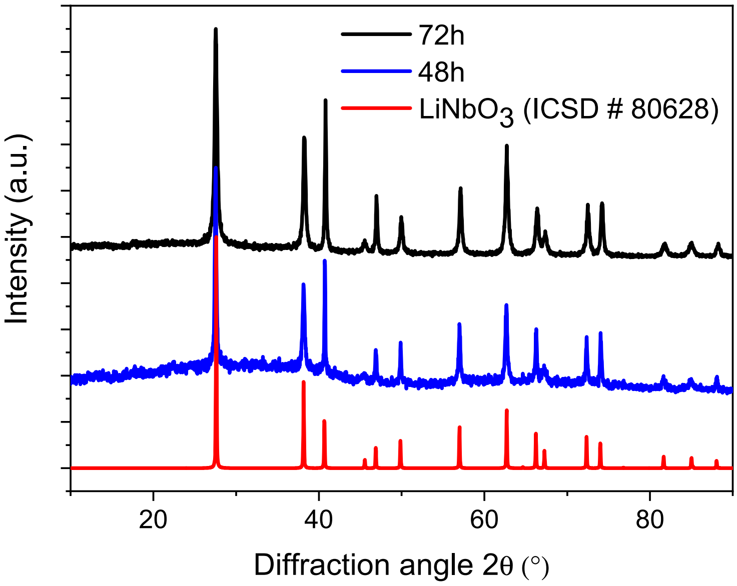

Figure S1. XRD diffraction patterns of LN nanopowders obtained after a thermal treatment at 230°C of the commercial precursor alone for a period extending from 48h to 72h. After 3 days, the absence of a significant amorphous contribution is attested from the almost flat baseline on the corresponding XRD profile.

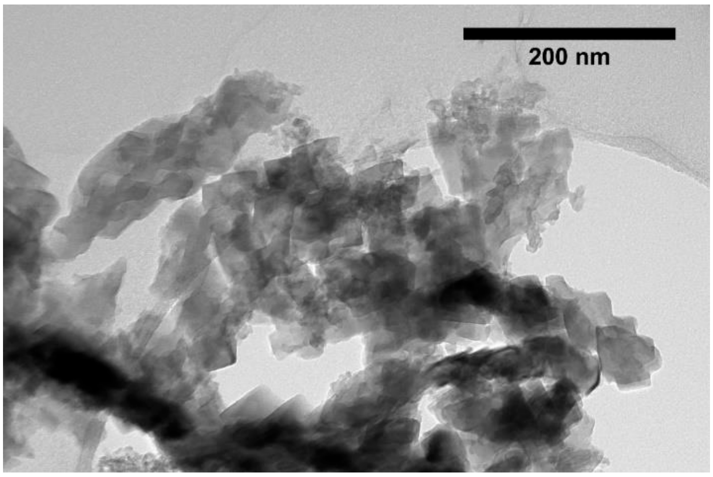

Figure S2. TEM image of LN nanoplatelets after dilution with ethanol of the precursor solution giving a molar concentration fixed at 0.077 M. Data treatment of the XRD profile (data not shown) results in a mean nanocrystal of S_{012} = 77 nm and an anisotropic factor f > 8.0.

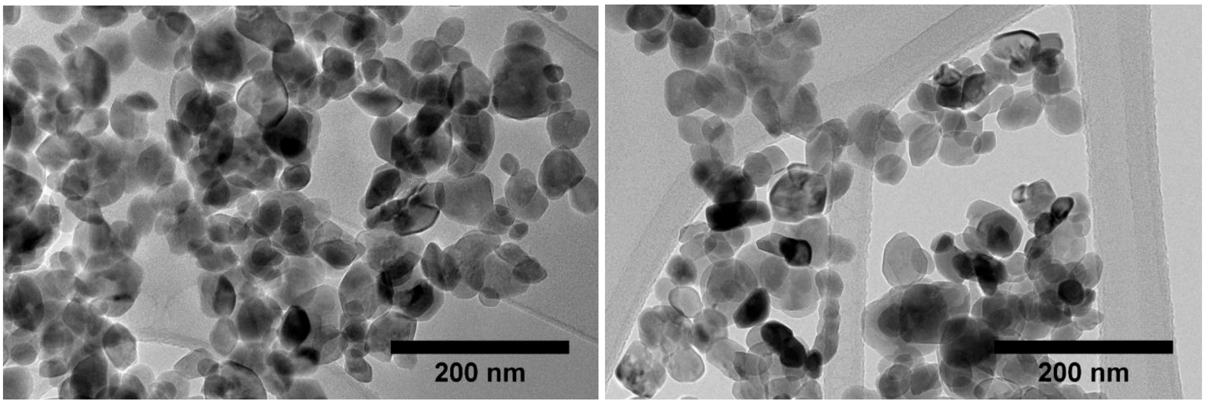

Figure S3. TEM images of LN nanocrystals at different filling fractions of the Teflon-cup. (Left) 2.5 mL of precursor and 1.9 mL of 1,4-Butanediol for a filling fraction at 19% and (Right) 5 mL of precursor and 3.8 mL of 1,4-Butanediol for a filling fraction at 38%. TEM images are very similar with the mean nanocrystal size S_{012} and anisotropic factor estimated at 30 nm and 2.5, respectively, in both cases. Effect of the autogenous pressure is negligible.

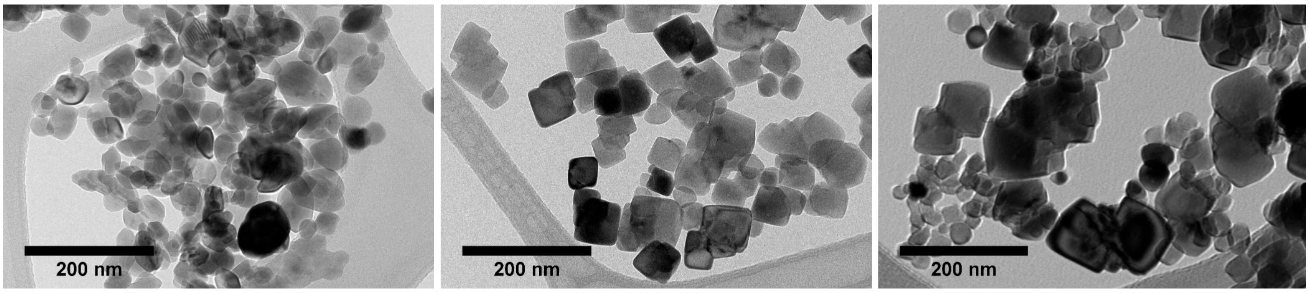

Figure S4. Influence of the ageing time on a precursor kept under ambient conditions for a composition of the reactive medium corresponding to 5 mL of precursor and 3.8 mL of 1,4-Butanediol. TEM images of $LiNbO_3$ nanocrystals for a synthesis performed with a freshly new precursor (Left) and after a few openings of the same precursor solution kept under ambient conditions for an ageing time of 1 month (Middle) and 3 months (Right). Note how the size and shape polydispersity is strongly affected in terms of facetization with the appearance of flattened cubic-shape nanocrystals when the precursor solution is not handled under an inert atmosphere.

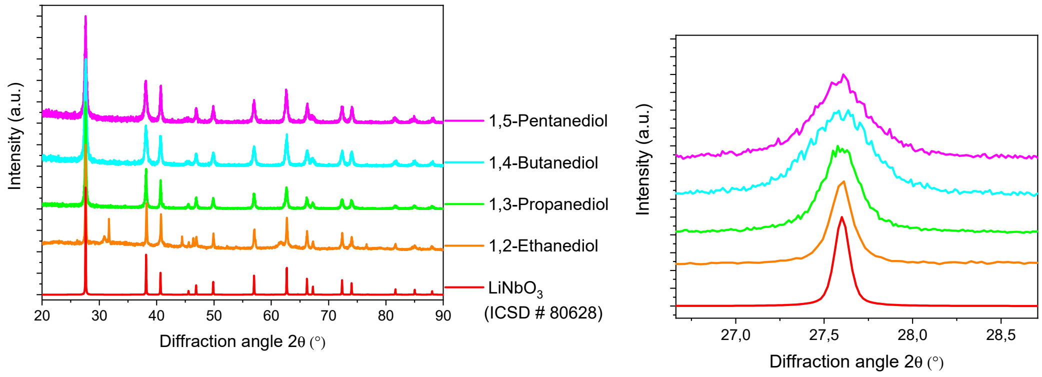

Figure S5. (Left) XRD diffraction patterns of LN nanopowders obtained after a thermal treatment at 230°C for 3 days of the commercial precursor with various co-solvents of increasing chain lengths. The molar ratio of glycol to ethanol is 0.5 in each case. (Right) Closer view at 2 ~ 27.5° of the (012) reflection showing a larger FWHM as long as the glycol chain is increased.

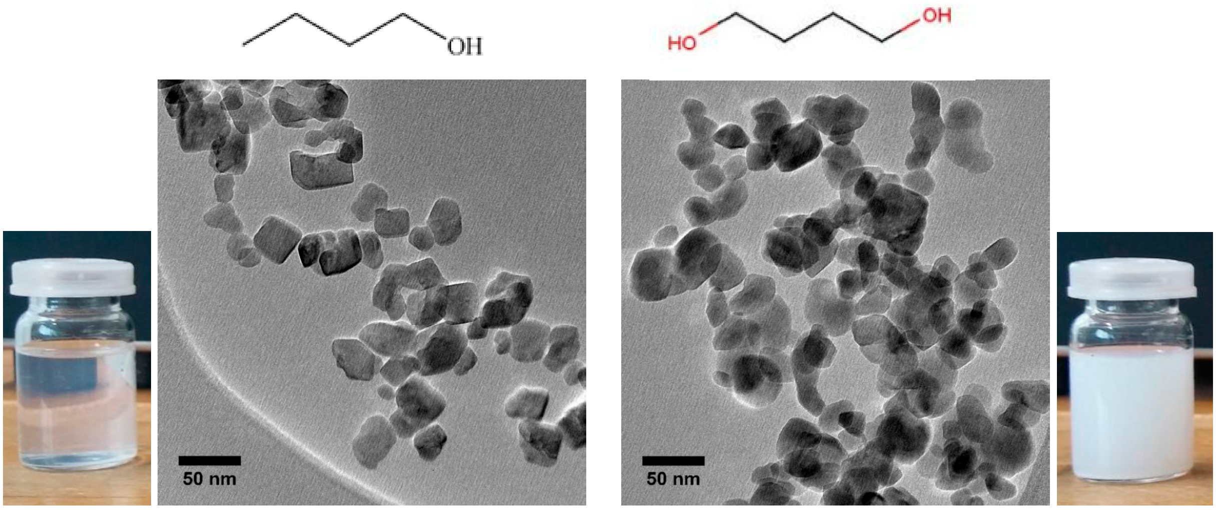

Figure S6. Comparison of the TEM images for $LiNbO_3$ nanocrystals produced at a molar ratio of 0.5 when Butanol (Left) and 1,4- Butanediol (Right) is added to 5 mL of the ethanolic precursor solution. Structure of each co-solvent is indicated in the upper panel and the corresponding optical images illustrate the absence of colloidal stability for the $LiNbO_3$ nanocrystals prepared with Butanol and dispersed at 0.1 mg/mL in ethanol after a period of 5 days.

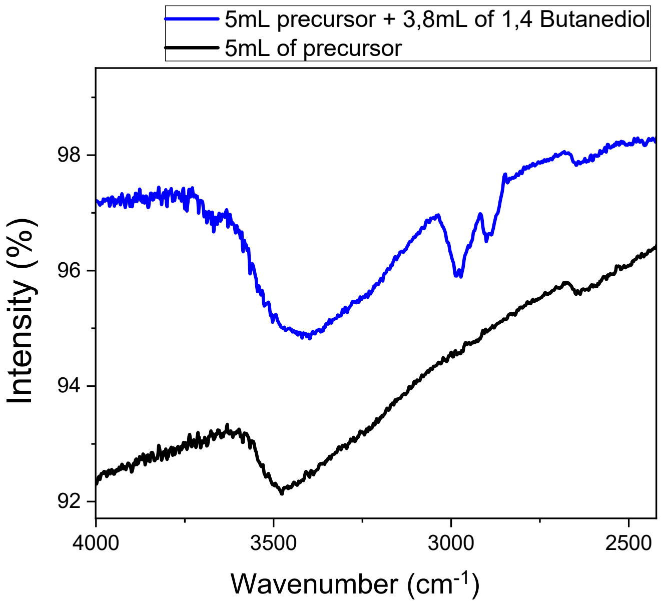

Figure S7. Comparison of FTIR spectra in the 2500-4000 cm^{-1} spectral range for $LiNbO_3$ nanocrystals obtained with and without 1,4-Butanediol. With the glycol, the higher amount of

hydroxyl groups and aliphatic -CH groups is visible from the large band at 3500 cm-1 and the two peaks below 3000 cm-1, respectively.

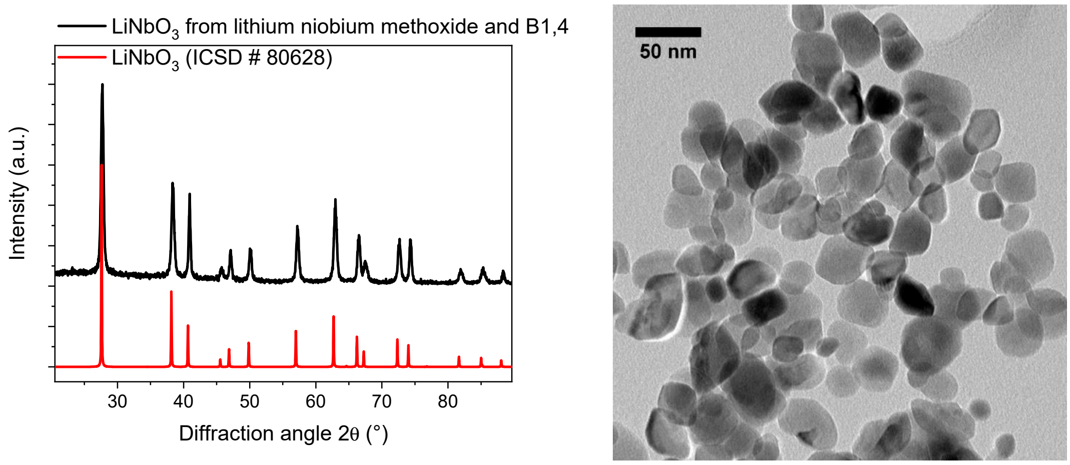

Figure S8. (Left) XRD diffraction pattern of LN nanocrystals obtained after a thermal treatment at 230°C for 3 days of 5mL of lithium niobium methoxide dissolved in methanol after addition of 4.9 mL of 1,4-Butanediol. (Right) Corresponding TEM image showing a nanocrystal morphology very similar to the one observed in Figure S6.

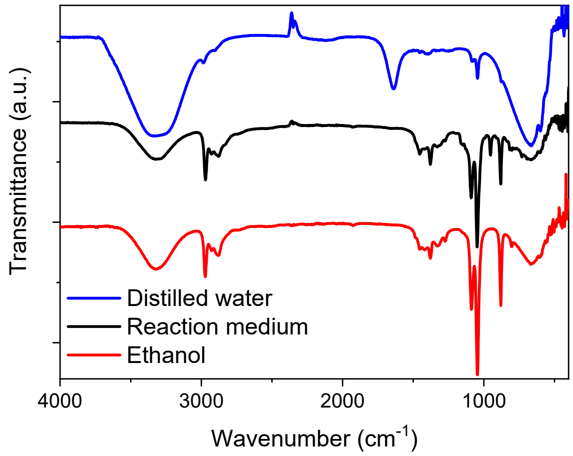

Figure S9. Comparison of the FTIR spectra of water and ethanol with the one of the reaction medium at the end of the 3-day solvothermal treatment does not evidence the characteristic absorption band of water at 1660 cm-1.

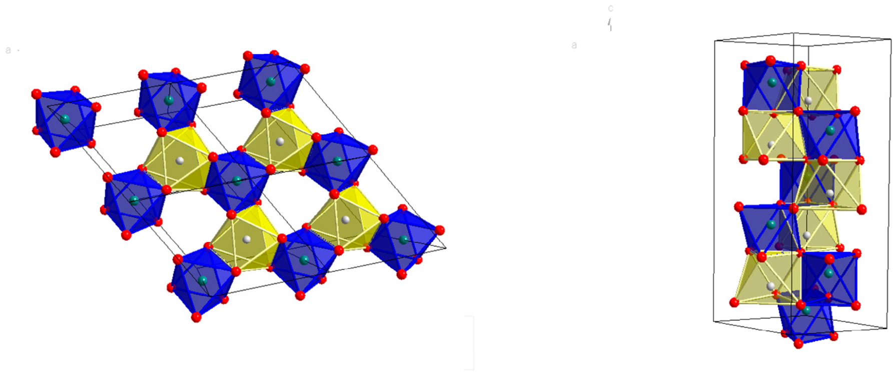

(**a**) (**b**)

Figure S10. (a) Arrangement of the edge-sharing octahedra surrounding the Nb^{5+} (in blue) and the Li+ ions (in Yellow) in the (002) crystalline plane. (b) Partial view of the face-sharing octahedra surrounding Nb^{5+} and Li+ along the polar direction whereas a isotropic corner-sharing octahedra arrangement is visible if only Li^{+} (or Nb^{5+}) ions are considered.
